# Optimization of Phenolic-Enriched Extracts from Olive Leaves via Ball Milling-Assisted Extraction Using Response Surface Methodology

**DOI:** 10.3390/molecules29153658

**Published:** 2024-08-02

**Authors:** Qixuan Xiang, Jingyi Wang, Kan Tao, Hu Huang, Yaping Zhao, Jinping Jia, Huijun Tan, Huailong Chang

**Affiliations:** 1 School of Chemistry and Chemical Engineering, Frontiers Science Center for Transformative Molecules, Shanghai Jiao Tong University, 800 Dong Chuan Road, Shanghai 200240, China; xiangqixuan@sjtu.edu.cn (Q.X.); ypzhao@sjtu.edu.cn (Y.Z.); jpjia@sjtu.edu.cn (J.J.); 2 Research and Development Department, Shanghai Chicmax Cosmetic Co., Ltd., 38th Floor, Global Harbor Tower B, No. 3300 North Zhongshan Road, Putuo District, Shanghai 200065, China; 60005925@kans.cn (J.W.); taokan@kans.cn (K.T.); huanghu@kans.cn (H.H.)

**Keywords:** olive leaves, phenolic compound, ball milling, response surface methodology, plant extraction

## Abstract

This study aims to extract phenolic-enriched compounds, specifically oleuropein, luteoloside, and hydroxytyrosol, from olive leaves using ball milling-assisted extraction (BMAE). Response surface methodology (RSM) and the Box–Behnken design (BBD) were used to evaluate the effects of the temperature, solvent-to-solid ratio, and milling speed on extraction recovery. The contents of the extract were determined by ultra-high-performance liquid chromatography–mass spectrometry (UPLC-MS) and converted to recoveries to evaluate the extraction efficiency. The optimal extraction conditions for oleuropein, luteoloside, and hydroxytyrosol were identified. Oleuropein had a recovery of 79.0% ± 0.9% at a temperature of 56.4 °C, a solvent-to-solid ratio of 39.1 mL/g, and a milling speed of 429 rpm. Luteoloside’s recovery was 74.6% ± 1.2% at 58.4 °C, 31.3 mL/g, and 328 rpm. Hydroxytyrosol achieved 43.1% ± 1.3% recovery at 51.5 °C, 32.7 mL/g, and 317 rpm. The reason for the high recoveries might be that high energy ball milling could reduce the sample size further, breaking down the cell walls of olive leaves, to enhance the mass transfer of these components from the cell to solvent. BMAE is displayed to be an efficient approach to extracting oleuropein, luteoloside, and hydroxytyrosol from olive leaves, which is easy to extend to industrial production.

## 1. Introduction

The olive plant (*Olea europaea* L.), native to the coastal regions of the Mediterranean, is renowned globally as a significant woody, oil-bearing economic tree species. Its distribution spans the European Mediterranean shores and the California region in the United States. Annually, olive cultivation results in the loss of millions of tons of leaves during pruning [1]. While some of these leaves serve as animal feed or bioenergy, the majority are either discarded or incinerated, leading to substantial resource waste and environmental degradation [2]. Olive leaves harbor a rich array of phenolic compounds, including iridoids, flavonoids, and simple phenols [3]. Notably, oleuropein, a major iridoid terpene, stands out for its high concentration and potent antioxidant properties due to its abundant catechol content [4,5]. Luteoloside is one of the key components of flavonoids, forming an oxidative heterocycle that exists in glycosylation form [6,7]. Additionally, hydroxytyrosol is classified as a simple phenolic compound and is an amphiphilic phenol with a phenylethanol structure [8]. Phenolic compounds contribute to the leaves’ pharmacological efficacy, exhibiting antioxidant [9], anti-inflammatory [10], antimicrobial [11], and anticarcinogenic [12] activities. These compounds find extensive applications in cosmetics, medicine, and health foods, presenting vast market potential [13].

Given the significant interest in extracting bioactive substances from olive leaves, various methods have been explored, including traditional methods like maceration extraction [14] and Soxhlet extraction [15], alongside modern approaches such as ultrasonic extraction [16], microwave extraction [17], and supercritical CO_2_ extraction [18]. Traditional extraction methods, despite their long-standing use, suffer from low efficiency, extensive solvent usage, and prolonged extraction times [19,20]. Conversely, newer techniques offer enhancements in efficiency, necessitating shorter extraction times and reduced solvent volumes. However, these methods are not without drawbacks: ultrasonic extraction and microwave extraction pose risks of degrading heat-sensitive compounds [21], whereas supercritical CO_2_ extraction, despite its effectiveness, incurs high equipment costs and struggles with extracting highly polar substances [22]. These limitations underscore the ongoing need for a novel extraction methodology that combines high efficiency, time and cost savings, and minimal environmental impact.

Ball milling extraction, a pioneering technology rooted in mechanochemical processes, offers substantial benefits [23]. It harnesses mechanical energy to enhance the mass transfer of raw materials through mechanisms such as friction, collision, extrusion, and shearing. Additionally, this technique can disrupt the structural integrity of plant tissues, diminishing particle size and breaking down cell walls. This facilitates a more efficient release of active compounds into the solvent [24,25]. Therefore, ball milling can significantly reduce extraction times and increase the solubility of bioactive substances in raw materials. However, the extraction of bioactive compounds from olive leaves utilizing ball-milling-assisted methods such as oleuropein, luteoloside, and hydroxytyrosol is rarely reported.

In this paper, the ball milling-assisted extraction (BMAE) technique is developed to extract phenolic compounds such as oleuropein, luteoloside, and hydroxytyrosol from olive leaves. The recoveries of various active compounds are assessed using ultra-high-performance liquid chromatography–mass spectrometry (UPLC-MS) to evaluate the extraction efficiency of BMAE for phenolic-enriched compounds. Aiming for optimal recoveries, response surface methodology (RSM) and Box–Behnken design (BBD) are applied to explore the influence of variables such as temperature, solvent-to-solid ratio, and milling speed on the extraction efficiency within the ranges of 40~60 °C, 20~40 mL/g, and 250~450 rpm, respectively. Recoveries of 79.0% ± 0.9% for oleuropein, 74.6% ± 1.2% for luteoloside, and 43.1% ± 1.3% for hydroxytyrosol are obtained at the optimal conditions. These results demonstrate that BMAE is an efficient technique for the extraction of valuable compounds from olive leaves.

## 2. Results and Discussion

### 2.1. Characterization of Compounds Extracted from Raw Material via Ultrasonic Extraction Using UPLC-MS

**Figure 1:**
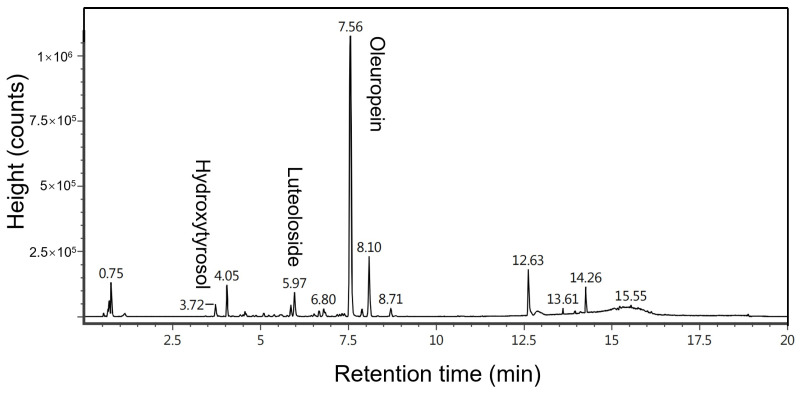
UPLC-MS chromatogram of ultrasonic extraction from olive leaf (raw material).

Natural dried olive leaves were crushed into powder under 40 mesh (Figure S1), followed by ultrasound-assisted extraction with a 70% methanol solution to study their chemical composition. The yield of olive leaf extract was 41.2 ± 0.7 wt%, which is considered to be a comparing standard for all the extractable compounds of the olive leaves in this text. The chemical analysis of these compounds is performed using ultra-high-performance liquid chromatography–mass spectrometry (UPLC-MS). The analysis chromatograms are displayed in Figure 1, which align with data reported in the literature [26,27]. Comparing Figure 1 with the chromatograms of standard samples (Figure S2), the oleuropein, luteoloside, and hydroxytyrosol of the extracts are identified by the retention times of 7.56 min, 5.97 min, and 3.72 min, respectively. Also, it can be calculated that the oleuropein content in the olive leaf raw material reaches 4.67% ± 0.09 wt%, the most abundant compound in the extract, followed by 0.46 ± 0.03 wt% of luteoloside and 0.56 ± 0.02 wt% of hydroxytyrosol. In summary, UPLC-MS was used to identify the compositions of the extracts and determine the total content of oleuropein, luteoloside, and hydroxytyrosol in the raw material, which can be taken as a basis for the calculation of their recoveries by BMAE.

### 2.2. Experimental Design and BMAE Results

**Figure 2:**
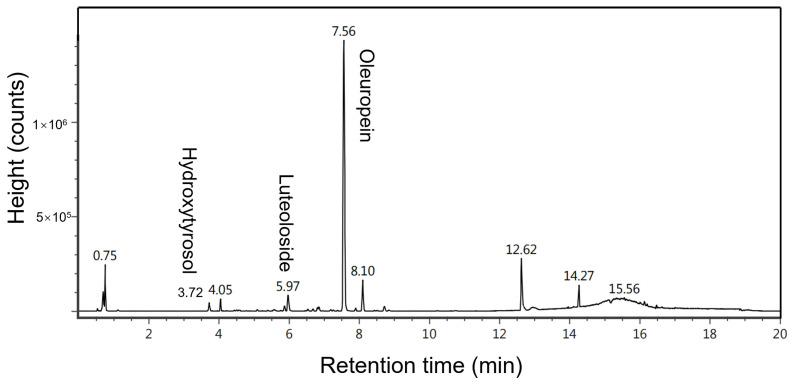
UPLC-MS chromatogram of olive leaf extract extracted by BMAE.

Response surface methodology (RSM), in conjunction with the Box–Behnken design (BBD), is utilized to assess the impacts of various factors on the extraction efficiency of oleuropein, luteoloside, and hydroxytyrosol from olive leaves by BMAE and to identify optimal extraction conditions. Building on the findings of prior research, we examined the influence of dependent variables, such as temperature (40~60 °C), solvent-to-solid ratio (20~40 mL/g), and milling speed (250~450 rpm), on the recoveries of these components during BMAE. A series of seventeen randomized trials was executed, including five repeats at a central point, to ensure the robustness of the data. The specified extraction parameters were set at a temperature of 50 °C, a solvent-to-solid ratio of 30 mL/g, a milling time of 1 h, and a milling speed of 350 rpm. The olive leaf extracts, derived under varying extraction conditions, underwent analysis and identification via UPLC-MS, which is shown in Figure 2. It can be seen that the chromatographic profile closely resembled that of the ultrasound extraction, and oleuropein was the predominant compound among the detected compounds. Moreover, the key substances of interest in this study, oleuropein, luteoloside, and hydroxytyrosol, matched the chromatographic signatures of standard compounds. This suggests that BMAE can be a viable technique for extracting active compounds from olive leaves.

**Figure 3:**
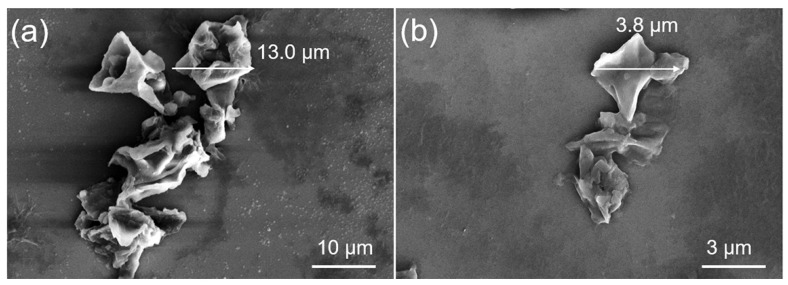
SEM images of olive leaf (**a**) before and (**b**) after BMAE.

The experimental design and the outcomes are detailed in Table 1, highlighting the recoveries observed in each trial. Regarding the experimental results, the recovery of oleuropein ranged from 55.5 ± 0.6% in run 3 to 77.0 ± 0.4% in run 15, the recovery of luteoloside ranged from 51.1 ± 0.3% in run 4 to 73.2 ± 0.2% in run 9, and the recovery of hydroxytyrosol ranged from 33.7 ± 0.5% in run 4 to 43.5 ± 0.2% in run 9. The results demonstrate that the BMAE is efficient in extracting significant amounts of oleuropein, luteoloside, and hydroxytyrosol from olive leaves. The reason stems from the process’s capability to thoroughly penetrate and break down, significantly reducing the particle size and disintegrating the cellular structure of the leaves and enhancing the mass transfer of the targeted compounds between the cell and solvent during grinding [28]. As illustrated in Figure 3, the particle size of olive leaves decreased from 10~50 µm to 1~5 µm before and after BMAE.

### 2.3. Model Fitting and Optimization of Recovery of Oleuropein

A comprehensive second-order polynomial model of the design is used to evaluate the recovery of oleuropein (response variable, *Y*) as a function of independent variables (*X*) and their interactions. Equation (1) expresses the statistical model as follows:

(1) $$Y=0.7374+0.0624\times A+0.0265\times B+0.0466\times C+0.0053\times AB-0.0035\times AC-0.0017\times BC-0.05\times A^{2}-0.0157\times B^{2}-0.0269\times C^{2}$$

where *Y* is the recovery of oleuropein (%) and *A*, *B*, and *C* are the variables for temperature, solvent-to-solid ratio, and milling speed, respectively.

Equation (1) reveals that the linear terms of the variables significantly influenced oleuropein’s recovery, with temperature (*A*) having the most pronounced effect, followed by milling speed (*C*) and solvent-to-solid ratio (*B*). The secondary effects of temperature (*A*^2^), solvent-to-solid ratio (*B*^2^), and milling speed (*C*^2^) also had significant negative statistical effects on the recovery of oleuropein.

Analysis of variance (ANOVA) was used to evaluate the accuracy of the model and the statistical significance of the input variables considered, and the analysis results of the recovery of oleuropein are shown in Table 2. The determination coefficient (R^2^), adjusted coefficient (R^2^_Adj_), and Fisher’s test (F-value, dependent on *p* < 0.05 (95%) confidence level) were critical in assessing the robustness of the RSM model. The model F-value of 73.78 signified its statistical significance, and a lack of fit F-value of 6.55 indicated that the lack of fit was not significant relative to the pure error. In addition, the value of R^2^ (0.9896) demonstrated a strong agreement between the observed data and the model, suggesting the model’s strong predictive capability. The signal-to-noise ratio, indicated by an adequate precision value of 27.6616, further validated the model’s reliability. In general, the model’s fitness and adequacy were assessed through its *p*-value and the lack of fit test, with *p*-values below 0.05 signifying the significance of the model terms. As shown in Table 2, the *p*-value of the complete quadratic model (0.0001, significant) and the lack of fit *p*-value (0.0505, not significant) confirmed the model’s adequacy in accurately representing the experimental data. Significant model terms (*A*, *B*, *C*, *A*^2^, *B*^2^, and *C*^2^) directly impacted oleuropein’s recovery, whereas interactions (*AB*, *AC*, *BC*) were found to be statistically non-significant, underscoring the nuanced relationship between the experimental conditions and the recovery of oleuropein.

**Figure 4:**
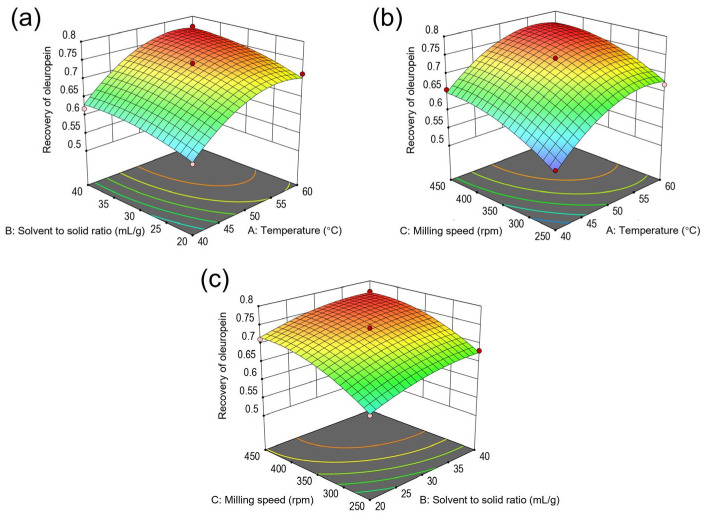
Response surface plots showing interactive effects of temperature, solvent-to-solid ratio, and milling speed on the recovery of oleuropein. (**a**) Interaction between temperature and solvent-to-solid ratio at a milling speed of 350 rpm; (**b**) interaction between temperature and milling speed at a solvent-to-solid ratio of 30 mL/g; (**c**) interaction between solvent-to-solid ratio and milling speed at a temperature of 50 °C.

The interaction of process parameters such as temperature, solvent-to-solid ratio, and milling speed on the extraction of oleuropein was investigated, and a three-dimensional (3D) surface response diagram was generated, as shown in Figure 4. The diagram’s slope sensitively indicates the degree of influence each factor had on the extraction outcome. A steeper slope signifies a greater impact on the response value, with the apex of the response surface plot marking the peak of pairwise interaction. Specifically, Figure 4a illustrates the dynamic between extraction temperature and solvent-to-solid ratio for oleuropein extraction. The results highlight that both the temperature and the solvent-to-solid ratio significantly affected oleuropein recovery, although their interaction did not present statistical significance (*p* > 0.05). An initial increase in oleuropein extraction was observed with rising temperature and solvent-to-solid ratio, followed by a decrease. The contour density revealed that the temperature had a more pronounced effect than the solvent-to-solid ratio on recovery. This phenomenon is attributed to higher temperatures enhancing solute solubility through increased vapor pressure and accelerated molecular activity, thus boosting extraction efficiency [29]. However, excessively high temperatures may lead to the degradation of phenolic compounds, diminishing the effectiveness of oleuropein extraction [30]. Further observations from Figure 4b indicate that increasing both extraction temperature and milling speed initially boosted extraction efficiency, which then diminished beyond certain levels. The contour density suggests that temperature’s impact on recovery slightly surpassed that of the milling speed. While increasing the milling speed augmented the mechanical force exerted by the milling balls, excessively high speeds caused the milling media to adhere closely to the mill’s inner surface, diminishing the mill’s ability to effectively disrupt the cell walls of the olive leaves and consequently lowering the recovery of the olive leaf extract [31,32]. Figure 4c demonstrates that oleuropein recovery was influenced by the solvent-to-solid ratio and milling speed, yet their interaction remained statistically insignificant (*p* > 0.05). An initial increase in oleuropein extraction was observed with rising extraction temperature and solvent-to-solid ratio, followed by a subsequent decrease. In essence, a higher solvent-to-solid ratio enhanced the concentration gradient, facilitating the solid–liquid mass transfer and thereby boosting the leaching rate of active compounds [33,34]. Nonetheless, an increased solvent-to-solid ratio would increase solvent expenses and the complexity of subsequent sample processing steps. Ultimately, the analysis indicates that the interplay among temperature, solvent-to-solid ratio, and milling speed does not significantly alter oleuropein recovery, suggesting that each factor independently influences the extraction efficiency.

In pursuit of maximizing oleuropein recovery, the optimal conditions as determined by the RSM model were implemented, yielding the highest response value at an extraction temperature of 56.4 °C, a solvent-to-solid ratio of 39.1 mL/g, and a milling speed of 429 rpm. The oleuropein recoveries observed in the experimental setup and as predicted by the RSM model were closely matched, at 79.0% ± 0.9% and 78.8%, respectively. This consistency between the experimental outcomes and model predictions affirms the RSM model’s accuracy. Therefore, the identified optimal extraction conditions were a temperature of 56.4 °C, a solvent-to-solid ratio of 39.1 mL/g, and a milling speed of 429 rpm, having been validated as the most effective for oleuropein extraction.

### 2.4. Model Fitting and Optimization of Recovery of Luteoloside

A full second-order polynomial model of the design was used to evaluate the recovery of luteoloside as a function of independent variables and their interactions. Equation (2) expresses the statistical model as follows:

(2) $$Y=0.7176+0.0616\times A+0.0269\times B-0.0382\times C-0.0235\times AB+0.0058\times AC+0.0042\times BC-0.0341\times A^{2}-0.0241\times B^{2}-0.0748\times C^{2}$$

where *Y* is the recovery of luteoloside (%) and *A*, *B*, and *C* are the variables for temperature, solvent-to-solid ratio, and milling speed, respectively.

The equation reveals that, for the linear terms of the three measured factors, temperature (*A*) and solvent-to-solid ratio (*B*) had significant positive effects on the recovery of luteoloside, while milling speed (*C*) showed a negative effect. Additionally, interactions between temperature solvent-to-solid ratio (*AB*), along with the quadratic effects of temperature (*A*^2^), solvent-to-solid ratio (*B*^2^), and milling speed (*C*^2^), had significant negative statistical effects on luteoloside recovery.

ANOVA for the quadratic model of luteoloside recovery, as detailed in Table 3, demonstrated the model’s significance with an F-value of 52.95 and a high level of accuracy, as indicated by a determination coefficient (R^2^) of 0.9855. The adjusted coefficient R^2^_Adj_ = 0.9669 indicated that 96.69% of the variation in the response could be explained by changes in temperature, solvent-to-solid ratio, and milling speed. Furthermore, the adequate precision of 21.7706 confirmed that the signal was sufficient. With a *p*-value for the complete quadratic model of < 0.0001 (*p* < 0.05, significant) and a lack of fit of 0.1359 (*p* > 0.05, not significant), the model was validated as a reliable representation of the experimental data. The significance of linear (*A*, *B*, *C*), quadratic (*A*^2^, *B*^2^, *C*^2^) and interactive model terms (*AB*) terms on luteoloside recovery was confirmed, whereas other interaction terms did not significantly affect luteoloside recovery.

**Figure 5:**
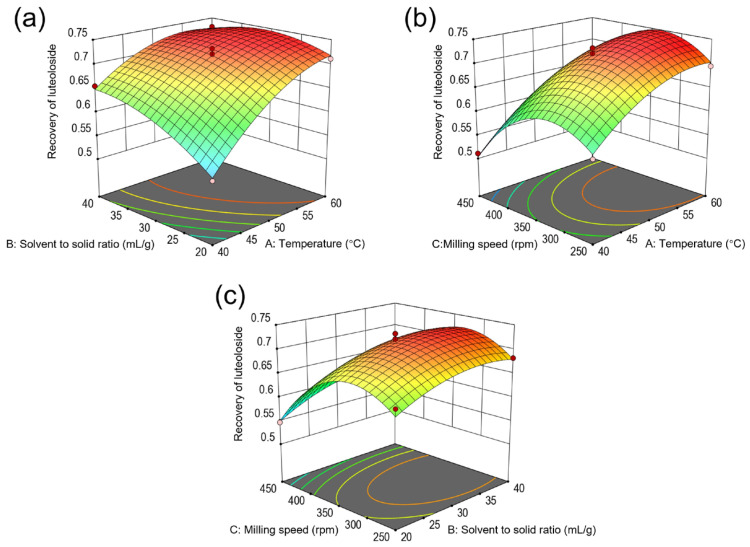
Response surface plots showing interactive effects of temperature, solvent-to-solid ratio, and milling speed on the recovery of luteoloside. (**a**) Interaction between temperature and solvent-to-solid ratio at a milling speed of 350 rpm; (**b**) interaction between temperature and milling speed at a solvent-to-solid ratio of 30 mL/g; (**c**) interaction between solvent-to-solid ratio and milling speed at a temperature of 50 °C.

The effects of temperature, solvent-to-solid ratio, and milling speed on luteoloside extraction were meticulously analyzed using a 3D surface response diagram, as depicted in Figure 5. The diagram’s slope precisely indicated the sensitivity of luteoloside recovery to changes in these parameters. In Figure 5a, a steep response surface curve illustrates the significant impact of the interplay between extraction temperature and solvent-to-solid ratio (*AB*) on luteoloside recovery. With the milling speed held constant at 350 rpm, and as the extraction temperature increased from 40 °C to 60 °C alongside a rise in solvent-to-solid ratio from 20 mL/g to 40 mL/g, luteoloside recovery initially improved, but eventually experienced a slight decline. This pattern suggests that temperature exerts a more dominant influence on luteoloside recovery compared to the solvent-to-solid ratio. Figure 5b further demonstrates that both temperature and milling speed significantly impacted luteoloside recovery when the solvent-to-solid ratio is maintained at 30 mL/g. However, the interaction between temperature and milling speed (*AC*) did not significantly influence luteoloside recovery (*p* > 0.05). This observation aligns with the findings presented in Figure 5c, which indicate that the interaction between solvent-to-solid ratio and milling speed (*BC*), similarly to the interaction showcased in Figure 5b, did not significantly alter luteoloside recovery. This comprehensive analysis underscores the nuanced effects of individual parameters and their interactions on the efficiency of luteoloside extraction via BMAE.

Guided by the RSM model, the optimal extraction parameters were identified as an extraction temperature of 58.4 °C, a solvent-to-solid ratio of 31.3 mL/g, and a milling speed of 328 rpm. At these parameters, the RSM model predicted an optimal luteoloside recovery of 74.9%. Experimental validation under these conditions confirmed a luteoloside recovery of 74.6% ± 1.2%, demonstrating the model’s efficacy and practical utility. Consequently, the optimal conditions for luteoloside extraction are a temperature of 58.4 °C, a solvent-to-solid ratio of 31.3 mL/g, and a milling speed of 328 rpm.

### 2.5. Model Fitting and Optimization of Recovery of Hydroxytyrosol

A full second-order polynomial model of the design was used to evaluate the recovery of hydroxytyrosol as a function of independent variables and their interactions. Equation (3) expresses the statistical model as follows:

(3) $$Y=0.4326+0.0129\times A+0.0010\times B-0.0106\times C+0.0153\times AB+0.0145\times AC+0.0017\times BC-0.0420\times A^{2}-0.0048\times B^{2}-0.0121\times C^{2}$$

where *Y* is the recovery of hydroxytyrosol (%) and *A*, *B*, and *C* are the variables for temperature, solvent-to-solid ratio, and milling speed, respectively.

The equation reveals that among the three evaluated factors, temperature (*A*) significantly enhanced the recovery of hydroxytyrosol, demonstrating a significant positive effect. In contrast, milling speed (*C*) negatively impacted hydroxytyrosol recovery. Interactions between temperature and solvent-to-solid ratio (*AB*), as well as temperature and milling speed (*AC*), positively influenced hydroxytyrosol recovery. However, the quadratic effects of temperature (*A*^2^) and milling speed (*C*^2^) exhibited significant negative effects on the recovery of hydroxytyrosol, indicating a more complex relationship between these factors and the extraction efficiency.

The ANOVA analysis for the secondary model regarding hydroxytyrosol recovery, detailed in Table 4, revealed critical insights into the model’s significance. A high F-value of 69.43 coupled with a *p*-value of <0.0001 underscored the regression model’s substantial significance. Furthermore, the determination coefficient R^2^ of the model was 0.9889, illustrating its capability to precisely delineate the relationship between influencing factors and hydroxytyrosol recovery. The regression equation not only serves as a tool for evaluating the model’s performance, but also demonstrates its robustness, as indicated by the lack of fit value of 0.0562, which signifies its statistical insignificance. In addition, the close alignment between the regression equation and experimental data is evidenced by an adjusted coefficient R^2^_adj_ = 0.9747, nearing unity. This alignment, alongside an adequate precision value of 26.7609, suggests the model’s robust predictive accuracy for the response surface values. Analysis of the model terms reveals that both linear (*A*, *C*) and quadratic terms (*A*^2^, *C*^2^), along with interaction terms (*AB*, *AC*), significantly influence hydroxytyrosol recovery. Conversely, other model terms were found not to significantly impact the recovery of hydroxytyrosol.

**Figure 6:**
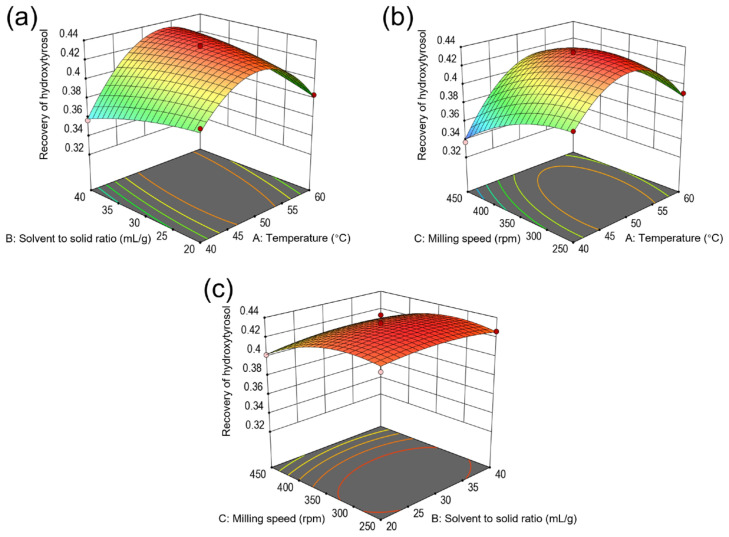
Response surface plots showing interactive effects of temperature, solvent-to-solid ratio, and milling speed on the recovery of hydroxytyrosol. (**a**) Interaction between temperature and solvent-to-solid ratio at a milling speed of 350 rpm; (**b**) interaction between temperature and milling speed at a solvent-to-solid ratio of 30 mL/g; (**c**) interaction between solvent-to-solid ratio and milling speed at a temperature of 50 °C.

Based on the regression equation, Figure 6 presents a 3D response surface diagram of the effects of temperature, solvent-to-solid ratio, and milling speed on hydroxytyrosol extraction. This diagram effectively captures the sensitivity of hydroxytyrosol recovery to variations in key parameters, highlighting that a steeper gradient signifies greater sensitivity. In Figure 6a, it can be observed that the solvent-to-solid ratio alone did not significantly impact hydroxytyrosol recovery. However, the combined effect of temperature and solvent-to-solid ratio (*AB*) markedly influenced hydroxytyrosol recovery. Notably, an increase in both temperature and solvent-to-solid ratio, while maintaining a constant milling speed of 350 rpm, boosted the extraction efficiency of hydroxytyrosol. Nonetheless, a decline in recovery was evident when the temperatures surpassed 50 °C. Similarly, Figure 6b showcases a pronounced impact of the interaction between temperature and milling speed (*AC*) on hydroxytyrosol recovery. As the temperature and milling speed rose from 40 °C and 250 rpm to 50 °C and 350 rpm, respectively, hydroxytyrosol recovery reached 43.5%. Yet, an additional increase in both factors significantly diminished the recovery. Conversely, the interaction between solvent-to-solid ratio and milling speed (*BC*), as depicted in Figure 6c, did not significantly affect hydroxytyrosol recovery. This analysis elucidates the complex interplay between specific extraction parameters and their collective impact on optimizing hydroxytyrosol extraction efficiency.

According to the model established in the experiment, the optimal extraction conditions for hydroxytyrosol were determined to be an extraction temperature of 51.5 °C, a solvent-to-solid ratio of 32.7 mL/g, and a milling speed of 317 rpm, predicting a hydroxytyrosol recovery of 43.6%. The actual experimental results yielded a hydroxytyrosol recovery of 43.1% ± 1.3%, demonstrating a minimal discrepancy with the predicted value. This close alignment underscores the effectiveness of the RSM in refining BMAE for hydroxytyrosol extraction. Consequently, the identified optimal conditions, a 51.5 °C extraction temperature, a 32.7 mL/g solvent-to-solid ratio, and a 317 rpm milling speed, were validated as the most efficient parameters for maximizing hydroxytyrosol recovery.

### 2.6. Optimal Extraction Conditions of Oleuropein, Luteoloside, and Hydroxytyrosol

The optimal conditions for BMAE of oleuropein, luteoloside, and hydroxytyrosol from olive leaves are presented in Table 5. The data indicate that increasing the milling speed and solvent-to-solid ratio positively impacts the recovery of oleuropein. In contrast, luteoloside exhibits stability with higher temperatures, showing no significant degradation. Hydroxytyrosol, being thermally unstable, demonstrates a relatively lower optimal extraction temperature. Furthermore, the predicted extraction recoveries align well with the experimental values, confirming the feasibility and reliability of the RSM.

## 3. Materials and Methods

### 3.1. Materials

Olive leaves were provided by Shanghai Zhongyi Daily Chemical Co., Ltd. (Shanghai, China). Ethanol was supplied from Shanghai Lingfeng Chemical Reagent Co., Ltd. (Shanghai, China). Standard compounds, including oleuropein, luteoloside, and hydroxytyrosol, with a purity of ≥98.0% were purchased from Chengdu Push Bio-technology Co., Ltd. (Chengdu, China). Nylon 66 membrane (0.22 μm) was provided by Haining De Lv New Material Technology Co., Ltd. (Haining, China). All reagents were employed without further purification.

### 3.2. Ultrasonic Extraction of Olive Leaves

An ultrasonic extraction method was employed as a standard method to extract the oleuropein, luteoloside, and hydroxytyrosol from olive leaves, with the extracted amount of each compound defined as their content in the olive leaves, respectively. Typically, naturally dried olive leaves were ground into 40-mesh powder using a mixer grinder and 5 g of the ground powder was placed in a beaker, to which 500 mL of a 70% methanol aqueous solution was added. Then, the beaker was put into an ultrasonic bath (280 W) to be extracted for 2 h. After that, the mixed solution was separated by filtration. The filter residue was extracted again using the same procedure and repeated two times. Each time, the filtrate solution was collected for analyzing the content later. This ultrasonic extraction procedure was performed three times, with the outcomes presented as the mean ± standard deviation.

### 3.3. Ball Milling Extraction of Olive Leaves

**Figure 7:**
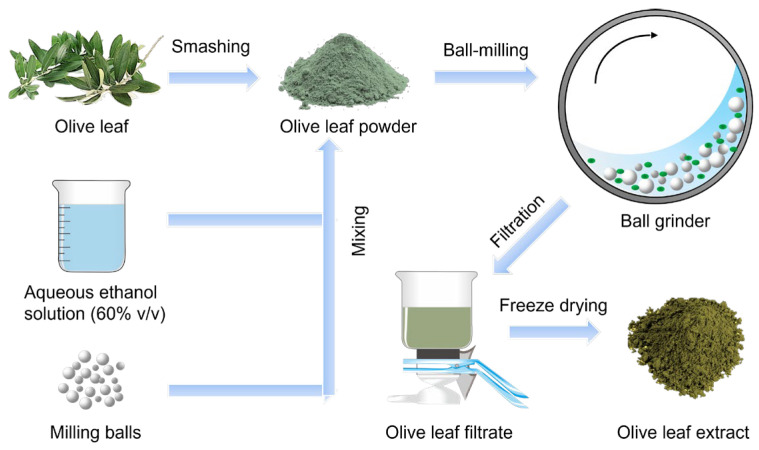
Schematic illustration of olive leaf extraction by BMAE.

Ball milling-assisted extraction (BMAE) was conducted using a mechanical planetary milling system (XQM-2, Changsha Tianchuang Powder Technology Co., Ltd., Changsha, China). Figure 7 displays a schematic illustration of olive leaf extraction by BMAE, in which naturally dried olive leaves were ground into 40-mesh powders first and then extraction was started using BMAE. In a typical extraction process, 6.67 g of olive leaf powder and 200 mL ethanol aqueous solution with a 60% volume fraction (solvent-to-solid ratio = 30 mL/g) were placed into an extraction vessel (made of ZrO_2_ and with a 500 mL capacity, provided by Changsha Tianchuang Powder Technology Co., Ltd.). Subsequently, 350 g ZrO_2_ balls of varying diameters were added to the vessel (Table S1). The total volume of olive leaves, ethanol aqueous solution, and ZrO_2_ balls was 50% of the vessel. The vessel was then sealed and mounted onto the planetary ball mill. BMAE was performed at a constant temperature of 50 °C and a milling speed of 350 rpm for 1 h. After that, the lid was opened to collect the mixed solution and vacuum filter to obtain the filtrate. The filtrated solution, as shown in Figure S3, was concentrated using a rotary evaporator and subsequently freeze-dried to obtain the final solid product, as illustrated in Figure S4.

The yield of olive leaf extract was determined according to Equation (4), as follows:

(4) $$Y\;\left(\%\right)=\frac{m_{1}}{m_{2}}\times 100\%$$

where $m_{1}$ = weight of olive leaf extract (g) and $m_{2}$ = weight of olive leaf raw materials (g).

The recoveries of oleuropein, luteoloside, and hydroxytyrosol in olive leaves were determined according to Equation (5):

(5) $$Recovery\;\left(\%\right)=\frac{m_{1}\times \omega_{1}}{m_{2}\times \omega_{2}}\times 100\%$$

where ω_1_ = mass fraction of the active substance in the extract (wt%) and ω_2_ = mass fraction of the active substance in raw material (wt%).

All the data are averages of triplicate measurements, expressed as mean ± standard deviation.

### 3.4. Design of Experiments and Response Surface Methodology

The response surface method (RSM) and Box–Behnken design (BBD) were employed to optimize the BMAE conditions for maximizing the recoveries of oleuropein, luteoloside, and hydroxytyrosol from olive leaves. This optimization involved three key factors, including temperature (*A*), solvent-to-solid ratio (*B*), and milling speed (*C*), each assessed at three levels, as detailed in Table 6. The recoveries of oleuropein, luteoloside, and hydroxytyrosol were the responses of the model. The objective was to identify the optimal condition combinations that would yield the highest recoveries for these substances, respectively, utilizing a statistical model through Design Expert software (version 13.0, Stat-Ease, Minneapolis, MN, USA).

Seventeen experimental runs focused on these parameters, with the central point test conducted five times to assess model fit. The response surface facilitated the exploration of interactions among the variables of temperature (*A*), solvent-to-solid ratio (*B*), and milling speed (*C*). A second-order polynomial equation was formulated based on the response data to model these interactions and determine the optimal extraction conditions. The general form of the mathematical quadratic response equation is shown in Equation (6) [35]:

(6) $$Y=\beta_{0}+\sum_{i=1}^{3}\beta_{i}X_{i}+\sum_{i=1}^{3}\beta_{ii}X_{i}^{2}+\sum_{i=1}^{2}\sum_{j=i+1}^{3}\beta_{ij}X_{i}X_{j}$$

Here, the response variable (*Y*) denotes the recoveries of oleuropein, luteoloside, and hydroxytyrosol in the olive leaf extract. The variables *X_i_* and *X_j_* represent the independent factors affecting the response. The variable *β_0_* denotes the model intercept, while *β_i_*, *β_ii_*, and *β_ij_* are model regression coefficients of the model for the linear, quadratic, and interaction effects, respectively, determined through the least squares method from the experimental data.

### 3.5. Analysis of Active Substances in Olive Leaves Using UPLC-MS

The active compounds in olive leaves were analyzed both qualitatively and quantitatively utilizing ultra-high-performance liquid mass spectrometry (Acquity UPLC I-class/VION IMS QTOF, Waters Technology Co., Ltd., Shanghai, China). The preparation method of the analytical sample was as follows: 20 mg olive leaf sample was mixed with 1 mL methanol solution and vortexed on a vortex mixer for 1 min. Subsequently, 100 μL of this solution was diluted with 900 μL methanol, vortexed for 1 min, and centrifuged at 12,000 rpm for 10 min for later ready analysis.

The UPLC was equipped with a BEH C18 column (2.1 × 100 mm) and maintained at a column temperature of 45 °C, with a flow rate of 0.4 mL/min and an injection volume of 1 μL. The mobile phases consisted of water with 0.1% formic acid (phase A) and acetonitrile with 0.1% formic acid (phase B). The solvent gradient conditions were 0 min, 5% B; 3 min, 20% B; 10 min, 100% B; 12 min, 100% B; 15 min, 5% B; 20 min, 5% B. In addition, mass spectrometry analysis was conducted in negative ion mode with a scan range of 50~1000 *m*/*z*. Optimal ESI-MS parameters included a capillary voltage of +2 kV, a cone hole voltage of 40 V, a desolvation temperature of 450 °C, a desolvation gas flow of 900 L/h, and a scan speed of 0.2 s.

The quantitative analysis of the extracted sample was performed using oleuropein, luteoloside, and hydroxytyrosol as standard compounds. Calibration curves for each of them were meticulously developed across six concentration levels, with ranges specified as 5.63 to 180 μg/mL for oleuropein (Figure S5), 0.78 to 25 μg/mL for luteoloside (Figure S6), and 1.02 to 32.5 μg/mL for hydroxytyrosol (Figure S7). The calibration curves demonstrated excellent linearity, with correlation coefficients exceeding 0.998, indicating precise and reliable quantification of these active compounds in olive leaves. All the data are the average of triplicate measurements, expressed as mean ± standard deviation.

### 3.6. Morphology Characterization

The morphology and structure of olive leaf powder before and after BMAE were examined by using a Field Emission Scanning Electron Microscope (FE-SEM, Nova Nano SEM 450, FEI, Williamsburg, VA, USA).

## 4. Conclusions

In summary, an innovative ball milling-assisted extraction (BMAE) technique was employed to simultaneously extract phenolic compounds such as oleuropein, luteoloside, and hydroxytyrosol from olive leaves. The influences of extraction temperature, solvent-to-solid ratio, and milling speed on the effective extraction were systematically optimized in terms of their recoveries using RSM and BBD to determine the optimal extraction conditions. For oleuropein, a temperature of 56.4 °C, a solvent-to-solid ratio of 39.1 mL/g, and a milling speed of 429 rpm yielded a recovery of 79.0% ± 0.9%. For luteoloside, the optimal conditions were 58.4 °C, 31.3 mL/g, and 328 rpm, resulting in a recovery of 74.6% ± 1.2%. Hydroxytyrosol was optimally extracted at 51.5 °C, 32.7 mL/g, and 317 rpm, with a recovery of 43.1% ± 1.3%. The BMAE technique was instrumental in reducing the particle size of olive leaves, thereby enhancing the release of active compounds by facilitating mass transfer between the material and the extraction solvent. Thus, BMAE can be an effective method to extract these compounds from olive leaves, which can be a potential way to produce these bioactive compounds from olive leaves. Moreover, the contents of oleuropein, luteoloside, and hydroxytyrosol can be further improved by additional separation and purification steps to obtain each compound individually.

## Figures and Tables

**Table 1:** Experimental values of the response variables at the design points.

	Independent Variable			Experimental Response		
Runs	Temperature (*A*) (°C)	Solvent-to-Solid Ratio (*B*) (mL/g)	Milling Speed (*C*) (rpm)	Recovery of Oleuropein (%)	Recovery of Luteoloside (%)	Recovery of Hydroxytyrosol (%)
1	40	20	350	58.4 ± 0.2	54.2 ± 0.5	39.1 ± 0.4
2	40	40	350	61.9 ± 0.1	65.5 ± 0.2	35.7 ± 0.2
3	40	30	250	55.5 ± 0.6	58.2 ± 0.1	39.2 ± 0.7
4	40	30	450	65.7 ± 0.3	51.1 ± 0.3	33.7 ± 0.5
5	50	20	250	61.7 ± 0.3	64.9 ± 0.6	42.2 ± 0.1
6	50	40	250	68.1 ± 0.4	68.2 ± 0.5	42.6 ± 0.4
7	50	20	450	71.2 ± 0.1	54.7 ± 0.1	40.2 ± 0.7
8	50	40	450	76.9 ± 0.3	59.7 ± 0.3	41.3 ± 0.3
9	50	30	350	73.7 ± 0.2	73.2 ± 0.2	43.5 ± 0.2
10	50	30	350	74.2 ± 0.3	72.1 ± 0.7	43.4 ± 0.6
11	50	30	350	74.3 ± 0.3	71.5 ± 0.2	42.9 ± 0.1
12	50	30	350	72.9 ± 0.1	71.2 ± 0.6	43.4 ± 0.2
13	50	30	350	73.6 ± 0.7	70.8 ± 0.4	43.1 ± 0.2
14	60	20	350	71.4 ± 0.2	71.1 ± 0.1	38.4 ± 0.5
15	60	40	350	77.0 ± 0.4	73.0 ± 0.2	41.1 ± 0.2
16	60	30	250	67.1 ± 0.2	69.5 ± 0.8	39.1 ± 0.7
17	60	30	450	75.9 ± 0.1	64.7 ± 0.5	39.4 ± 0.1

**Table 2:** ANOVA for response surface quadratic model for the recovery of oleuropein.

Source	Sum of Squares	DF	Mean Square	F-Value	*p*-Value	Inference
Model	0.0702	9	0.0078	73.78	<0.0001	significant
*A*—Temperature	0.0311	1	0.0311	294.45	<0.0001	significant
*B*—Solvent-to-solid ratio	0.0056	1	0.0056	53.15	0.0002	significant
*C*—Milling speed	0.0174	1	0.0174	164.52	<0.0001	significant
*AB*	0.0001	1	0.0001	1.04	0.3411	not significant
*AC*	0	1	0	0.4635	0.5179	not significant
*BC*	0	1	0	0.1159	0.7435	not significant
*A* ^2^	0.0105	1	0.0105	99.38	<0.0001	significant
*B* ^2^	0.001	1	0.001	9.82	0.0165	significant
*C* ^2^	0.0031	1	0.0031	28.93	0.001	significant
Residual	0.0007	7	0.0001	-	-	-
Lack of Fit	0.0006	3	0.0002	6.55	0.0505	not significant
Pure Error	0.0001	4	0	-	-	-
Cor Total	0.0709	16	-	-	-	-
Std. Dev.	0.0103	-	-	-	-	-
Mean	0.6938	-	-	-	-	-
C.V. %	1.48	-	-	-	-	-
R^2^	0.9896	-	-	-	-	-
R^2^_Adj_	0.9762	-	-	-	-	-
R^2^_Pred_	0.8586	-	-	-	-	-
Adeq. Precision	27.6616	-	-	-	-	-
PRESS	0.01	-	-	-	-	-

**Table 3:** ANOVA for response surface quadratic model for the recovery of luteoloside.

Source	Sum of Squares	DF	Mean Square	F-Value	*p*-Value	Inference
Model	0.0838	9	0.0093	52.95	<0.0001	significant
*A*—Temperature	0.0304	1	0.0304	172.84	<0.0001	significant
*B*—Solvent-to-solid ratio	0.0058	1	0.0058	32.87	0.0007	significant
*C*—Milling speed	0.0117	1	0.0117	66.59	<0.0001	significant
*AB*	0.0022	1	0.0022	12.57	0.0094	significant
*AC*	0.0001	1	0.0001	0.7524	0.4145	not significant
*BC*	0.0001	1	0.0001	0.411	0.5419	not significant
*A* ^2^	0.0049	1	0.0049	27.77	0.0012	significant
*B* ^2^	0.0024	1	0.0024	13.85	0.0074	significant
*C* ^2^	0.0236	1	0.0236	134.02	<0.0001	significant
Residual	0.0012	7	0.0002	-	-	-
Lack of Fit	0.0009	3	0.0003	3.36	0.1359	not significant
Pure Error	0.0003	4	0.0001	-	-	-
Cor Total	0.085	16	-	-	-	-
Std. Dev.	0.0133	-	-	-	-	-
Mean	0.6551	-	-	-	-	-
C.V. %	2.02	-	-	-	-	-
R^2^	0.9855	-	-	-	-	-
R^2^_Adj_	0.9669	-	-	-	-	-
R^2^_Pred_	0.8277	-	-	-	-	-
Adeq. Precision	21.7706	-	-	-	-	-
PRESS	0.0146	-	-	-	-	-

**Table 4:** ANOVA for response surface quadratic model for the recovery of hydroxytyrosol.

Source	Sum of Squares	DF	Mean Square	F-Value	*p*-Value	Inference
Model	0.0126	9	0.0014	69.43	<0.0001	significant
*A*—Temperature	0.0013	1	0.0013	65.86	<0.0001	significant
*B*—Solvent-to-solid ratio	8 × 10^−6^	1	8 × 10^−6^	0.3973	0.5485	not significant
*C*—Milling speed	0.0009	1	0.0009	44.85	0.0003	significant
*AB*	0.0009	1	0.0009	46.2	0.0003	significant
*AC*	0.0008	1	0.0008	41.77	0.0003	significant
*BC*	0	1	0	0.6084	0.461	not significant
*A* ^2^	0.0074	1	0.0074	369.74	<0.0001	significant
*B* ^2^	0.0001	1	0.0001	4.82	0.0642	not significant
*C* ^2^	0.0006	1	0.0006	30.36	0.0009	significant
Residual	0.0001	7	0	-	-	-
Lack of Fit	0.0001	3	0	6.12	0.0562	not significant
Pure Error	0	4	6.3 × 10^−6^	-	-	-
Cor Total	0.0127	16	-	-	-	-
Std. Dev.	0.0045	-	-	-	-	-
Mean	0.4049	-	-	-	-	-
C.V. %	1.11	-	-	-	-	-
R^2^	0.9889	-	-	-	-	-
R^2^_Adj_	0.9747	-	-	-	-	-
R^2^_Pred_	0.8514	-	-	-	-	-
Adeq. Precision	26.7609	-	-	-	-	-
PRESS	0.0019	-	-	-	-	-

**Table 5:** Recoveries of oleuropein, luteoloside, and hydroxytyrosol under optimal extraction conditions.

	Temperature (*A*) (°C)	Solvent-to-Solid Ratio (*B*) (mL/g)	Milling Speed (*C*) (rpm)	Predicted Recovery	Experimental Recovery
Oleuropein	56.4	39.1	429	78.8%	79.0% ± 0.9%
Luteoloside	58.4	31.3	328	74.9%	74.6% ± 1.2%
Hydroxytyrosol	51.5	32.7	317	43.6%	43.1% ± 1.3%

**Table 6:** Experimental values and coded levels of the independent variables used for the rotatable central composite design.

		Code Level		
		Low	Center	High
Independent variable	Code variable	−1	0	+1
Temperature (°C)	*A*	40	50	60
Solvent-to-solid ratio (mL/g)	*B*	20	30	40
Milling speed (rpm)	*C*	250	350	450

## Data Availability

The data presented in this research are available upon request from the corresponding author.

## Supplementary Materials

The following supporting information can be downloaded at: https://www.mdpi.com/article/10.3390/molecules29153658/s1, Figure S1: Olive leaf raw materials (a) before and (b) after crushing. Figure S2: UPLC-MS chromatogram of standard hydroxytyrosol, luteolin, and oleuropein. Figure S3: Olive leaf extract solution. Figure S4: Olive leaf extract powder. Figure S5: Calibration curve of oleuropein standards. Figure S6: Calibration curve of luteoloside standards. Figure S7: Calibration curve of hydroxytyrosol standards. Table S1: The ratio of milling balls: Diameter and wt% of grinding ZrO_2_ balls used.
