# Supplementary material for: Optimization of Phenolic-Enriched Extracts from Olive Leaves via Ball Milling-Assisted Extraction Using Response Surface Methodology
Source: Molecules. 2024 Aug 2;29(15):3658. doi: 10.3390/molecules29153658 (PMC11314388; doi:10.3390/molecules29153658)
Supplement: Supplementary file 1 [file molecules-29-03658-s001.zip › molecules-3088400-supplementary.pdf]

## Supporting information

# Optimization of Phenolic-Enriched Extracts from Olive Leaves via Ball Milling-Assisted Extraction Using Response Surface Methodology

**Qixuan Xiang [1,†], Jingyi Wang [2,†], Kan Tao [2], Hu Huang [2], Yaping Zhao [1], Jinping Jia [1], Huijun Tan [1,*] and Huailong Chang [2,*]**

[1]School of Chemistry and Chemical Engineering, Frontiers Science Center for Transformative Molecules, Shanghai Jiao Tong University, 800 Dong Chuan Road, Shanghai 200240, PR China

[2]Research and Development Department, Shanghai Chicmax Cosmetic Co. Ltd., 38th Floor, Global Harbor Tower B, No. 3300 North Zhongshan Road, Putuo District, Shanghai 200065, China

*Corresponding author: Huijun Tan, Huailong Chang

Tel.: +86 21 54743274; E-mail: sophie93@sjtu.edu.cn; 60006496@kans.cn

†These authors contributed equally to this work.

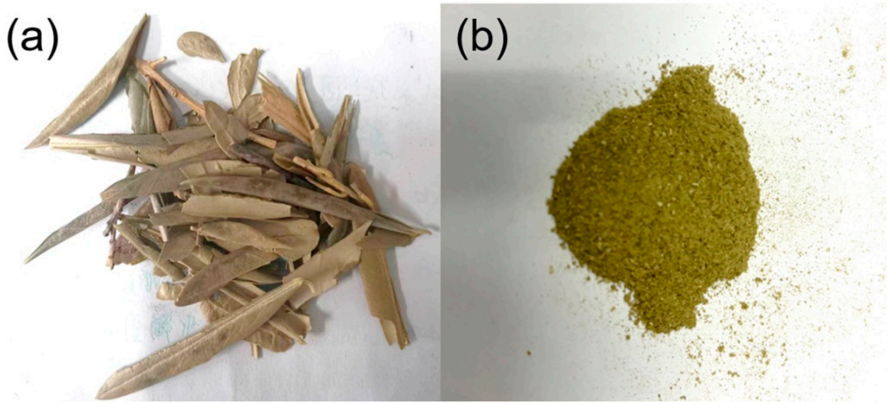


**Figure S1.** Olive leaf raw materials **(a)** before and **(b)** after crushing.

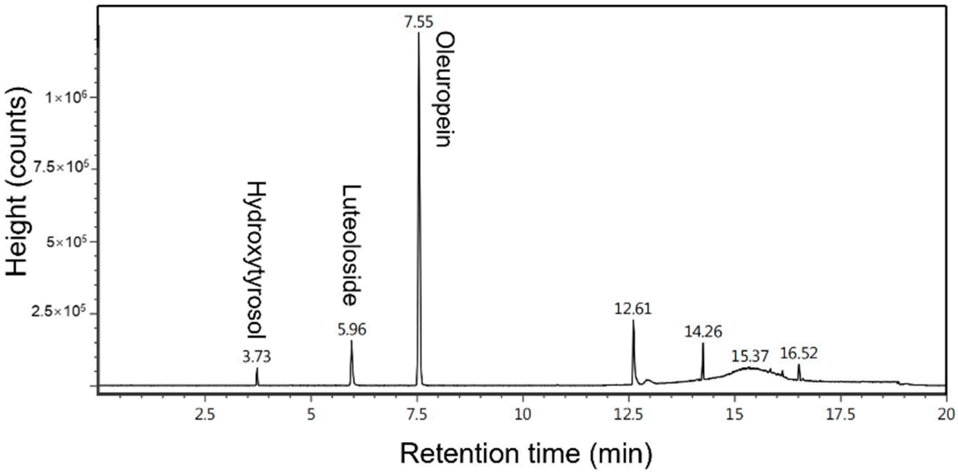


**Figure S2.** UPLC-MS chromatogram of standard hydroxytyrosol, luteolin and oleuropein.

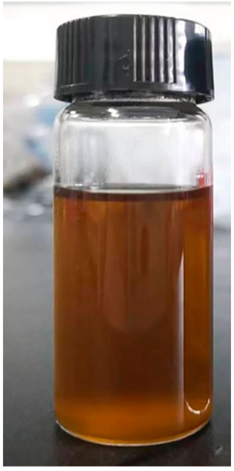

**Figure S3.** Olive leaf extract solution.

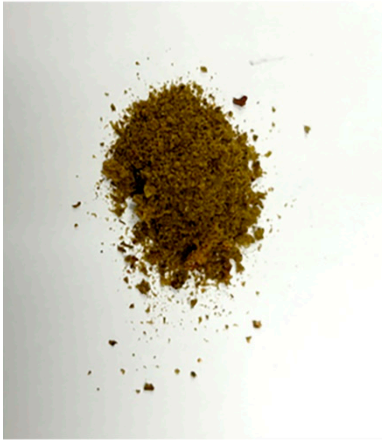

**Figure S4.** Olive leaf extract powder.

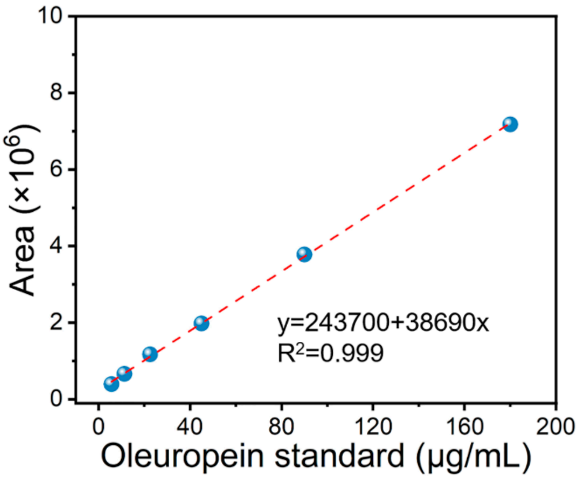


**Figure S5.** Calibration curve of oleuropein standards.

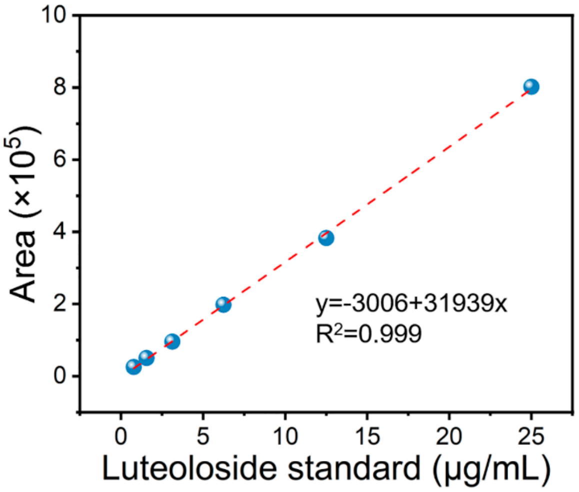


**Figure S6.** Calibration curve of luteoloside standards.

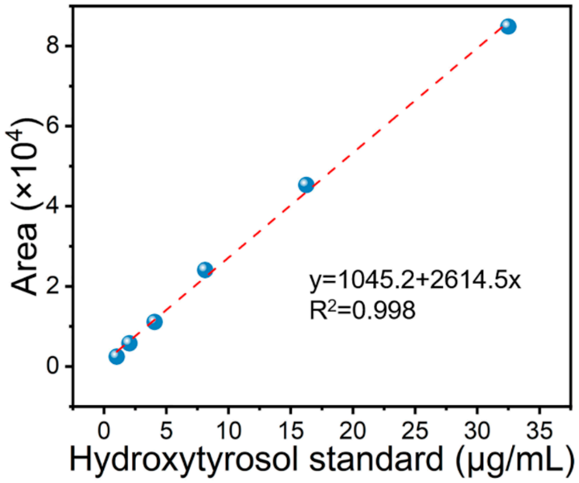


**Figure S7.** Calibration curve of hydroxytyrosol standards.

**Table S1.** The ratio of milling balls: Diameter and wt% of grinding $ZrO_2$ balls used.

| Ball diameter (mm) | Weight (g) | Weight ratio (%) |
|---|---|---|
| 15 | 35 | 10 |
| 12 | 53 | 15 |
| 10 | 70 | 20 |
| 8.5 | 105 | 30 |
| 5 | 88 | 25 |
